# Very early onset of coronary artery aneurysm in a 3-month infant with Kawasaki disease: a case report and literature review

**DOI:** 10.1186/s13052-023-01478-9

**Published:** 2023-06-04

**Authors:** Wenyan Jiao, Li Wei, Fuyong Jiao, Dorina Pjetraj, Jianying Feng, Jvyan Wang, Carlo Catassi, Simona Gatti

**Affiliations:** 1 Department of Psychology, Shaanxi Provincial People’s Hospital, Xi’an, China; 2 Shaanxi Kawasaki Disease Diagnosis and Treatment Center, Xi’an, China; 3 Department of Pediatrics, Polytechnic University of Marche, Via Filippo Corridoni 11, 60123 Ancona, Italy

**Keywords:** Kawasaki disease, Coronary artery aneurysm, Thrombosis, Children, Case report

## Abstract

**Background:**

Kawasaki disease (KD) is a medium vessel vasculitis, of unknown etiology, typically presenting in children younger than 5 years of age. Prolonged fever (at least five days) is a major clinical criterion of KD, while cardiac involvement may occur in up to 25% of patients, generally in the second week of the disease.

**Case presentation:**

We describe the case of KD developing in a 3-month infant, with an early occurrence of coronary artery aneurysm after only 3 days of fever, complicated by thrombosis, requiring aggressive treatments.

**Conclusions:**

Time of development of cardiac complications can be different in young infants with KD and both diagnostic criteria and treatment indications should be individualized in this class of age.

## Background

Kawasaki disease (KD), also known as mucocutaneous lymph node syndrome (MCLS), was first reported by Nagasaki Fuzuo in 1967. KD is an acute systemic vascular inflammatory disease, occurring in young children (under 5 years of age), which mainly involves systemic medium vessels, with coronary artery involvement being the most serious complication [1]. Classical clinical manifestations include fever, rash, cervical non-suppurative lymphadenopathy, conjunctival congestion, diffuse congestion of oral mucosa, strawberry tongue, palmoplantar erythema, and hand-foot edema. Atypical or incomplete presentation can make the diagnosis of KD more difficult and delay treatment. Presentation of KD in young infants (less than 6 months) can pose further diagnostic challenges for the lack of typical symptoms in this subgroup of patients and therapeutic difficulties, due to the increased risk of cardiac involvement [2]. In untreated children, the occurrence of severe cardiovascular complications can be as high as 20–25%, thus making KD the primary cause of acquired heart disease in children in developed regions.

We present the case of an infant with a short history of fever and rapid development of coronary damage, in order to alert pediatricians to investigate for coronary involvement also in atypical cases of suspected KD, especially in very young infants.

## Case presentation

A 3-month old male child was admitted to the Emergency Department of Shaanxi Provincial People' s Hospital for a 2-h history of low-grade fever (peak 37.7 °C) and maculo-papular rash. At admission there were no other symptoms or relevant clinical signs and no history of previous treatments.

At physical examination the child was febrile, with mild tachycardia (145/min), normal breathing rate (36/min) and blood pressure (68/55 mmHg). A maculo-papular slightly prominent rash was evident on the trunk and limbs. Lymph nodes were not enlarged, there were no signs of conjunctivitis or mucositis. Neurological, abdominal, and cardiovascular examinations were all normal. Pulmonary auscultation revealed presence of coarse crepitations. Weight was 6.2 kg and length was 64.7 cm (both appropriate for age).

Routine blood test showed relative neutrophilia (75%, mean value for age: 32%), with normal white blood cells count (12.73 × 10^9^/L), hemoglobin level (133 g/L) and platelet count (199 × 10^9^/L), increased high-sensitivity C-reactive protein (> 5 mg/L) and C-reactive protein (23.1 mg/dL). A naso-pharyngeal swab for Coronavirus-19 tested negative.

**Fig. 1:**
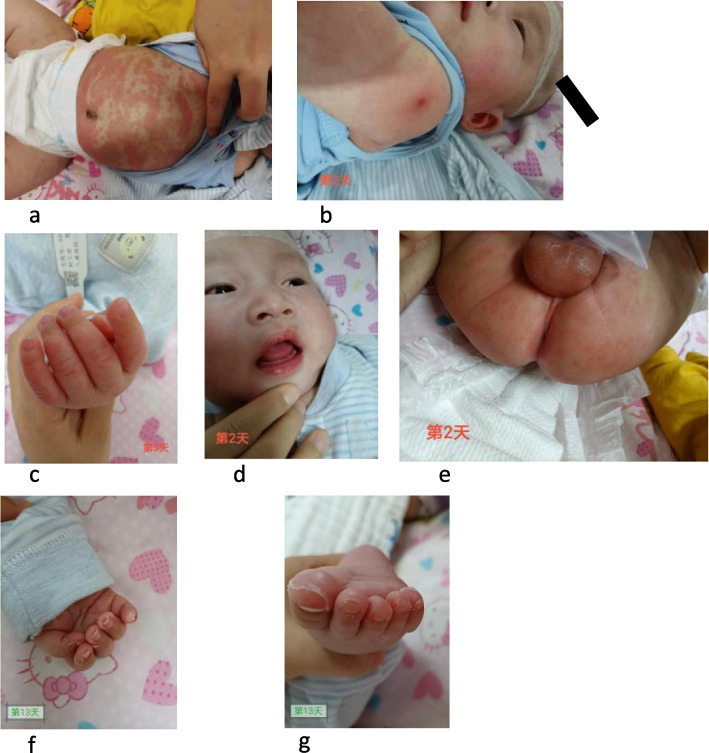
Initial and late clinical signs of disease course. **a** and **b**. Sprinkled skin rash on the abdomen, trunk and limbs **c**. edema of the fingers **d**. bilateral conjuntivitis, red and dry lips, red bayberry tongue **e**. perianal redness **f**. hands desquamation **g**. feet desquamation

**Fig. 2:**
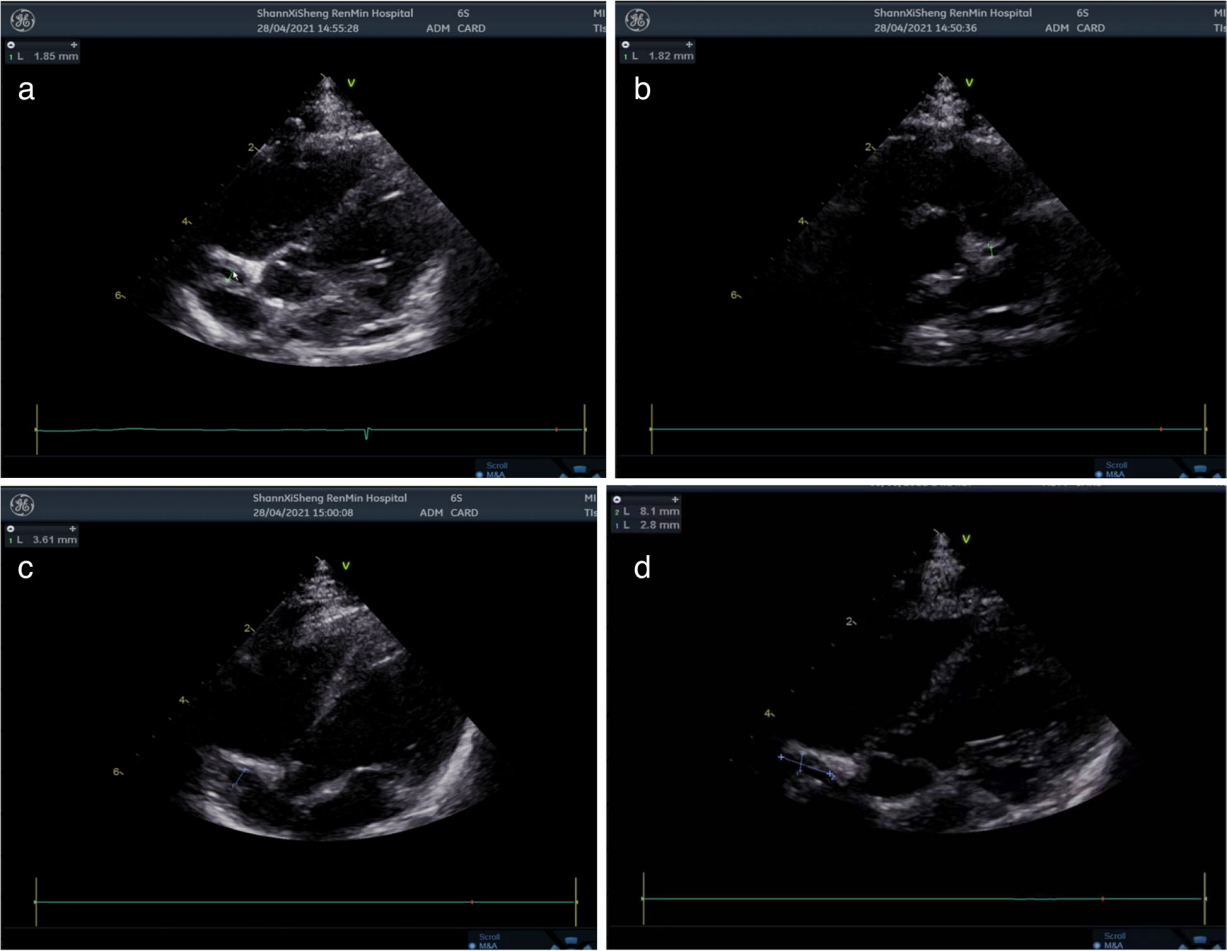
Echocardiography on day 4 of course of disease. **a** Apical five chamber view showing dimensions of the right coronary artery. **b** Parasternal short axis view showing left anterior descending branch. **c** Apical five chamber view showing dimensions of the proximal right coronary artery. **d** Apical five chamber view showing the intramural throbosis

**Fig. 3:**
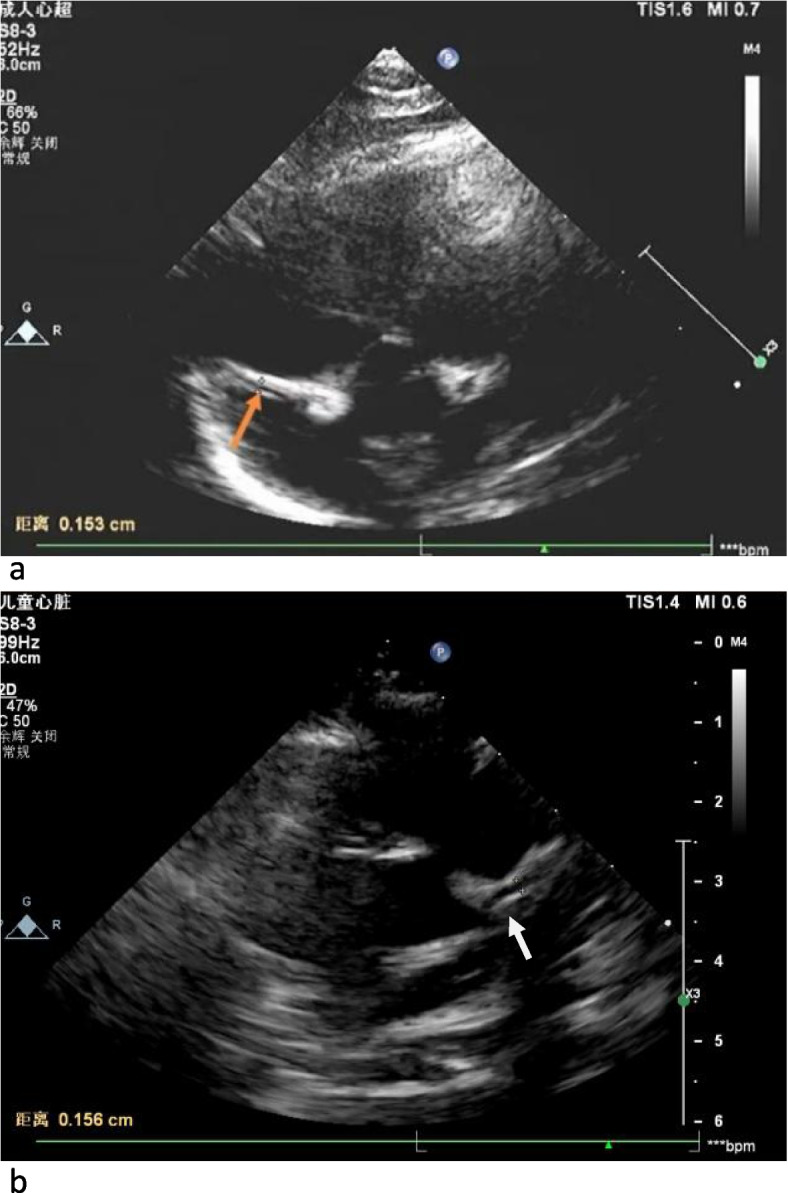
Echocardiography on the 46th day of disease course. Ultrasound examination of the heart showed that coronary artery dilation was improved. **a** Five chamber view showing dimensions of right coronary artery. **b** Parasternal short axis view showing left coronary artery (4a and 4b)

The child was admitted and an empiric treatment with IV cefotaxin was started. On the 2^nd^ day of admission, the child was still febrile (39.5 °C) with increased irritability, abdominal distension and cold extremities and rash spread to the face and trunk. Swelling and redness of the hands and feet, lips and oral mucosa redness (strawberry tongue), bilateral conjunctivitis and perioral redness became evident, as shown in Fig. 1 a-e. Repeated blood tests revealed raised pro-calcitonin (7.676 ng/mL), slightly increased liver enzymes (ALT 89 U/L, AST 44 U/L) and mild hypoalbuminemia (30.6 g/L). Leukocyturia (white blood cells 3 +) and proteinuria (582.6 ul) were evident at urine analysis. The main infective causes were excluded, including TORCH, EBV, gastrointestinal infection (normal stool culture and viral investigations), upper respiratory tract infection (negative pharyngeal swab). COVID serology was negative, such as blood and urine cultures. Mild signs of pulmonary inflammation were found on chest X-ray. Antibiotic treatment was switched to ceftriaxone to increase the spectrum and glycyrrhizin was introduced for liver protection. Three days after admission, blood analyses showed increased leukocytosis and clotting derangement (Fibrinogen: 6.08 g/L, Fibrin degradation products: 25.6 mg/L, D-dimer 9.62 mg/L). An echocardiogram showed normal diameter of the left main coronary artery (1.9 mm, z-score: 1.17, LCA/AO = 0.21), left anterior descending branch dilatation (1.8 mm, z-score: 2.08, LAD/AO = 0.20), and presence of a coronary aneurysm at the proximal right coronary artery (3.6 mm, z-score: 6.98, RCA/AO = 0.40) [3], with intra-aneurysmatic non-occlusive thrombosis (Fig. 2 a-d). Despite the young age and the short history of symptoms, a diagnosis of KD was considered. After obtaining parental informed consent, treatment with intravenous human immunoglobulin (IVIG, 2 g/Kg) was immediately given to block the immune response process and reduce further coronary damage. At the same time, a combination of anticoagulant therapies including oral aspirin (100 mg bid) and dipyridamole (12.5 mg bid) was prescribed. Despite a transient relief of symptoms for one day, 24 h after IVIG administration, fever, rash and the other clinical manifestations and biochemical signs recurred. A second dose of IVIG (2 g/Kg) was infused, along with intravenous steroids (methylprednisolone, 2 mg/kg). Following this treatment, symptoms rapidly disappeared, with an increase in platelet count to (770 × 10^9^/L) and evidence of hands and feet desquamation (Fig. 1f and g). A second echocardiography repeated after one week showed stable findings. The patient was clinically stable and therefore discharged from hospital on day 14 after a 3^rd^ echocardiography showing a mild improvement (LCA z-score: 0.86, LAD z-score: 0.43, RCA z-score: 6.01) and a regular follow-up was conducted after discharge according to the management plan (formulated by the Kawasaki Disease Cooperation Group of the Pediatric Branch of the Chinese Medical Association and the relevant guidelines of Japan and the United States) [1, 4, 5]. Two weeks post-discharge, the echocardiography showed no significant improvement in the coronary artery lesion and the thrombosis measures. Liver enzymes were significantly increased (ALT 119 U/L, AST 54 U/L). Therefore, warfarin treatment (0.09 mg/kg) was introduced, maintaining prothrombin international normalized ratio (INR) between 2 and 3 and aspirin and dypiridamole were stopped. Liver enzymes normalized after one week. At 4 weeks from discharge (day 46) the child was clinically well, weight was 6.7 kg and length was 66 cm. The echocardiography did not show evidence of thrombosis and dilatation of coronary artery was markedly improved ((LCA 1.6 mm, z-score: 0.12, LAD: 1.8 mm, z-score: 1.95, RCA 1.6 mm, z-score: 0.79). The results of echocardiography on the 46^th^ day of disease are shown in Fig. 3. At final follow-up (7 weeks post-discharge), the echocardiogram showed LCA 2.0 mm, z-score 1.47and RCA 1.6 mm,z-score: 0.79. Platelet count progressively normalized during observation and other blood tests were repeatedly normal (Table 1).

**Table 1:** Full blood count results at different time points

	**WBC(× 10**^**9**^**/L)**	**NEU(× 10**^**9**^**/L)**	**RBC(× 10**^**12**^**/L)**	**HB(g/L)**	**PLT(× 10**^**9**^**/L)**
Day 1	12.73	7.5	4.81	133	199
Day 4	12.76	8.25	3.67	96	277
Day 8	16.66	6.4	3.41	89	367
Day 14	10.23	03.2	3.5	94	770
Day 26	8.87	1.21	4.24	111	568
Day 41	7.66	0.94	4.13	109	412
Day 46	5.73	0.7	4.18	109	225

## Discussion and conclusions

Kawasaki disease is an acute, systemic vasculitis involving small and medium arteries, typically affecting children under 5 years of age. Since the first report of KD disease in 1976, the incidence has been rising. The highest incidence is reported in Japan with recent data (2016) indicating an incidence of 309/100,000 [6]. In China KD is now being increasingly recognized, in fact while in 2008–2012 the incidence in the area of Shanghai was 30.3–71.9/100,000 children (< 5 years) [7], more recently (2013–2017) in the same region it was increased to 68.8–107.3/100,000 children [8]. In developed countries, KD has become the most common cause of acquired heart disease [1], replacing rheumatic heart disease.

We have reported a peculiar case of incomplete KD in a young infant with a very early onset of a medium coronary aneurysm and a favorable outcome. The classical diagnostic criteria of KD, including fever plus one of the major findings can present at different times, making the diagnosis very challenging at an early stage. Particularly in young children (< 6 months of age) symptoms of KD can be unspecific, atypical or the clinical picture can be incomplete [2, 9]. In very young infants KD can be easily misdiagnosed as sepsis, urinary infection or non-specific inflammatory disease.

The most serious cardiac complications including coronary aneurysms, pericardial effusion, left ventricular enlargement and mitral insufficiency, typically develop in the second week of disease [1]. Demonstration of coronary abnormalities at echocardiography confirms the diagnosis, even if clinical criteria are not complete. Due to the use of IVIG and progress in treating KD, the incidence of coronary artery complications in KD has decreased from 20% to about 5%. Despite the progress there is still 0.1% of pediatric patients developing giant coronary artery aneurysm (GCAA) [6], mainly in the middle right coronary artery and the anterior descending left coronary artery, leading to a high incidence of cardiovascular events, mortality and poor prognosis [8].

The risk of coronary dilatations and aneurysm seems to be increased in younger children (< 6 months) and mortality is higher compared with older KD patients [10–12]. It remains unclear whether the major severity of KD in young infants is the consequence of the diagnostic difficulties and consequently delayed diagnosis or whether it is secondary to specific age-related factors.

Studies have reported that the time range of CAA reaching the maximum diameter in children with Kawasaki disease is 11 to 87 days, and the median is 35 days (n = 195) [13]. Our case presented with an exceptional early onset of coronary dilatation (3 days of illness) suggesting the need of maintaining an high index of suspicion of KD in a young infant even in the absence of a complete clinical picture.

Despite the description of a higher risk of coronary abnormalities and cardiac risks in younger infants with KD, it’s not clear whether the effect of a younger age influences also the timing of onset of coronary damage. In the study by Salgado et al [14] not only coronary dilation was more common but also was diagnosed earlier in the course of the disease in younger than in older infants. Similar results are described in the study by Moreno et al. [15], where a higher baseline z-score and z-max and a higher prevalence of dilatations and aneurysms were already present at first echocardiogram in the youngest infants (< 6 months) compared to older children.

Timely treatment of KD with IVIG (within seven days), can significantly reduce the risk of coronary artery aneurysms. Our patient was treated with IVIG on day 4 of fever, however there was recurrence of symptoms requiring further treatments. At present, the mechanism of gamma globulin resistance is not completely clear, and it is hypothesized to be related to genetic factors, in particular to the polymorphism of Fcγ receptor [16]. In our patient, after early infusion of the second dose of gamma globulin combined with glucocorticoids, symptoms were relieved and there was no evidence of cardiac complications. The use of steroids along with IVIG as initial treatment of high-risk KD is still controversial. Results from a meta-analysis found that a front-line combination of steroids with IVIG in high-risk KD patients reduced the rate of coronary artery abnormalities [17] with data coming mostly from Japan.It remains to be elucidated whether this treatment choice should be applied in high-risk populations outside of Japan. According to the AHA guidelines, steroids and other treatments (anti-TNF alpha) remain a second line choice, in cases of IVIG resistance. We treated our patient with a second dose of IVIG along with steroids (2 mg/kg) with a full clinical response. Recent studies have also found that the incidence of coronary artery lesions (CAL) varies significantly depending on the timing of IVIG treatment, suggesting that early use of IVIG is an effective method to inhibit systemic inflammation and prevent CAL [18].

Aspirin has anti-inflammatory and antiplatelet effects, and it is a coadjuvant therapy in KD. The time and dose of aspirin are still controversial [1, 5]. Recent studies have shown that compared with low-dose, medium and high doses of aspirin have no advantages in preventing CAL [19, 20]. In our case we selected a medium-dose aspirin regimen. The lack of improvement of the thrombosis at 2 weeks follow-up and the parallel increase in liver enzymes convinced us to introduce an anticoagulant medication. Warfarin is currently recognized as a safe anticoagulant for the prevention and treatment of coronary artery thrombosis. It has the advantages of good oral absorption, rapid action, long half-life, and simple schedule (once a day). In clinical practice, dose adjustment should be carried out in combination with the monitoring of the thrombosis and the severity of coronary artery lesions. Studies have shown that warfarin has a positive effect on the prognosis of coronary artery lesions and thrombosis in children with KD complicated with CAA [21], although the risk of thrombosis remains significant (14% in patients treated with warfarin) [21].

In conclusion this case-report suggests that coronary artery damage can appear very early in the course of KD and reminds clinicians to perform cardiac ultrasound examination at an early stage in cases of suspected KD. If cardiac damage is confirmed, prompt treatment with IVIG should be initiated and close follow-up is necessary, especially in young infants.

## Data Availability

The datasets used and/or analyzed during the current study are available from the corresponding author on reasonable request.

## Abbreviations

KD: Kawasaki disease
MCLS: Mucocutaneous lymph node syndrome
IVIG: Intravenous human immunoglobulin
INR: Prothrombin international normalized ratio
GCAA: Giant coronary artery aneurysm
CAL: Coronary artery lesions
